# Comparison of Acute Kidney Injury in Patients with COVID-19 and Other Respiratory Infections: A Prospective Cohort Study

**DOI:** 10.3390/jcm10112288

**Published:** 2021-05-25

**Authors:** Matthias Diebold, Tobias Zimmermann, Michael Dickenmann, Stefan Schaub, Stefano Bassetti, Sarah Tschudin-Sutter, Roland Bingisser, Corin Heim, Martin Siegemund, Stefan Osswald, Gabriela M. Kuster, Katharina M. Rentsch, Tobias Breidthardt, Raphael Twerenbold

**Affiliations:** 1 Clinic for Transplantation Immunology and Nephrology, University Hospital Basel, University of Basel, 4031 Basel, Switzerland; matthias.diebold@usb.ch (M.D.); stefan.schaub@usb.ch (S.S.); 2 Department of Intensive Care Medicine, University Hospital Basel, University of Basel, 4031 Basel, Switzerland; martin.siegemund@usb.ch; 3 Division of Internal Medicine, University Hospital Basel, University of Basel, 4031 Basel, Switzerland; Stefano.Bassetti@usb.ch (S.B.); tobias.breidthardt@usb.ch (T.B.); 4 Department of Clinical Research, University of Basel, 4031 Basel, Switzerland; Sarah.Tschudin@usb.ch; 5 Division of Infectious Disease & Hospital Epidemiology, University Hospital Basel, University of Basel, 4031 Basel, Switzerland; 6 Emergency Department, University Hospital Basel, University of Basel, 4031 Basel, Switzerland; roland.bingisser@usb.ch (R.B.); heim.c@gmx.ch (C.H.); 7 Department of Cardiology, University Hospital Basel, University of Basel, 4031 Basel, Switzerland; stefan.osswald@usb.ch (S.O.); gabriela.kuster@usb.ch (G.M.K.); raphael.twerenbold@usb.ch (R.T.); 8 Department of Laboratory Medicine, University Hospital Basel, University of Basel, 4031 Basel, Switzerland; katharina.rentsch@usb.ch; 9 Department of Cardiology and University Center of Cardiovascular Science, University Heart and Vascular Center Hamburg, 20246 Hamburg, Germany

**Keywords:** acute kidney injury, COVID-19, pneumonia, respiratory tract infection, SARS-CoV-2, mortality

## Abstract

Previous studies have indicated an association between coronavirus disease 2019 (COVID-19) and acute kidney injury (AKI) but lacked a control group. The prospective observational COronaVIrus-surviVAl (COVIVA) study performed at the University Hospital, Basel, Switzerland consecutively enrolled patients with symptoms suggestive of COVID-19. We compared patients who tested positive for SARS-CoV-2 with patients who tested negative but with an adjudicated diagnosis of a respiratory tract infection, including pneumonia. The primary outcome measure was death at 30 days, and the secondary outcomes were AKI incidence and a composite endpoint of death, intensive care treatment or rehospitalization at 30 days. Five hundred and seven patients were diagnosed with respiratory tract infections, and of those, 183 (36%) had a positive PCR swab test for SARS-CoV-2. The incidence of AKI was higher in patients with COVID-19 (30% versus 12%, *p* < 0.001), more severe (KDIGO stage 3, 22% versus 13%, *p* = 0.009) and more often required renal replacement therapy (4.4% versus 0.93%; *p* = 0.03). The risk of 30-day mortality and a composite endpoint was higher in patients with COVID-19-associated AKI (adjusted hazard ratio (aHR) mortality 3.98, 95% confidence interval (CI) 1.10–14.46, *p* = 0.036; composite endpoint aHR 1.84, 95% CI 1.02–3.31, *p* = 0.042). The mortality risk was attenuated when adjusting for disease severity (aHR 3.60, 95% CI 0.93–13.96, *p* = 0.062). AKI occurs more frequently and with a higher severity in patients with COVID-19 and is associated with worse outcomes.

## 1. Introduction

Acute kidney injury (AKI) has been shown to be a common complication in patients with coronavirus disease 2019 (COVID-19), with a reported incidence ranging from 7% to 49% [1,2,3,4,5]. The occurrence of AKI in patients with COVID-19 is known to be associated with a poor prognosis and prolonged disease [4,6]. However, most studies on AKI in patients with COVID-19 to date were of a retrospective nature, missed follow-up information or did not have an adequate control group to put them into perspective [4,5,6]. The COVID-19 pandemic is unique in its extent and widespread impact in modern times, and healthcare systems all around the world are being met with an unprecedented workload. Therefore, understanding the burden of COVID-19 and associated AKI is of great importance. The aim of this study was to compare AKI in patients with COVID-19 to patients with other respiratory tract infections and to investigate the differences in terms of the characteristics and outcomes.

## 2. Materials and Methods

### 2.1. Study Design, Population and Inclusion Criteria

The prospective, observational, COronaVIrus-surviVAl (COVIVA, ClinicalTrials.gov NCT04366765) study included unselected patients aged 18 years and older presenting consecutively with clinically suspected or confirmed SARS-CoV-2 infection to the emergency department (ED) of the University Hospital in Basel, Switzerland between 23 March and 31 May 2020. All patients underwent nasopharyngeal SARS-CoV-2 swab testing. Patients were considered COVID-19-positive if one or multiple SARS-CoV-2 PCR swab tests performed at the day of ED presentation or within 14 days prior to or post-ED presentation were positive, in combination with the clinical signs and symptoms. The remainder of patients with negative SARS-CoV-2 swab test results were considered as controls. All participating patients or their legally authorized representatives consented by signing a local general consent form. This study was conducted according to the principles of the Declaration of Helsinki and was approved by the local ethics committee (Ethics Commission of Northwestern and Central Switzerland (EKNZ) identifier 2020-00566).

The authors designed the study, gathered and analyzed the data according to the STROBE guidelines, vouched for the data and analysis, wrote the paper and decided to submit it for publication. These related files are shown on Supplementary Materials.

### 2.2. Adjudication of Final Diagnosis

To determine the final diagnosis that led to the index ED presentation, trained physicians reviewed all the available medical data, including 30-day post-discharge follow-up information. The predefined main categories included, but were not limited to, COVID-19; non-SARS-CoV-2 infections (e.g., respiratory, gastrointestinal and urogenital); cardiovascular disease (acute coronary syndrome, congestive heart failure and pulmonary embolism); other pulmonary noninfectious diseases (e.g., asthma and chronic obstructive pulmonary disease) and neurologic disease (e.g., stroke and seizure). For this analysis, we compared patients with COVID-19 to patients with a final adjudicated diagnosis of a respiratory tract infection other than COVID-19, including viral infections and bacterial pneumonia.

### 2.3. Clinical Assessment

All patients underwent a thorough clinical assessment by the treating ED physician according to the local standard operating procedure. The vital parameters, including heart rate, blood pressure, oxygen saturation and respiratory rate, were assessed in every patient at the time of ED presentation. The patients’ management was left to the discretion of the attending physician in accordance with the local standard operating procedures and current clinical practice guidelines.

### 2.4. Blood Sampling

Blood samples were routinely drawn in every patient at the time of ED presentation. Timing and type of subsequent laboratory measurements during the hospital stay were left to the discretion of the treating physicians and were not part of this study protocol.

### 2.5. Acute Kidney Injury

AKI was defined according to the serum creatinine criteria of the 2012 KDIGO clinical practice guidelines for acute kidney injury as an increase in serum creatinine to ≥1.5 times the baseline, which is known or presumed to have occurred within the prior 7 days, or an increase in serum creatinine by ≥0.3 mg/dL (≥26.5 µmoL/L) within 48 h [7]. The baseline steady-state kidney function was determined using electronic medical records of the last 6 months prior to the index hospitalization or the nadir of the hospitalization. In the absence of preadmission data and serial creatinine values, the baseline creatinine was imputed to 75 mL/min/1.73 m^2^, as per the KDIGO AKI guidelines [7]. AKI was graded using the KDIGO criteria. The urine output criterion was not used to define AKI, because the urine output was not regularly documented. The glomerular filtration rate was estimated using the CKD Epidemiology Collaboration (CKD-EPI) creatinine equation [8]. AKI at the day of presentation was defined as community-acquired AKI.

Renal recovery was defined as a decline of 33% from the peak serum creatinine levels compared to the discharge serum creatinine levels, as previously proposed [9]. Patients with KDIGO grade I also met the definition of renal recovery by a decline in the serum creatinine levels of ≥26.5 µmol. Patients requiring renal replacement at discharge did not meet the definition of renal recovery.

## 3. Follow-Up

Thirty days after discharge, patients were contacted by telephone or in written form by research physicians/study nurses, and information about their current health, hospitalizations and adverse events were obtained, guided by a predefined set of questions and standardized item checklists. Records of hospitals and primary care physicians, as well as national death registries, were screened for additional information, if applicable.

### 3.1. Outcomes

The primary outcome was all-cause death at 30 days. The secondary outcomes were AKI during index hospitalization; renal recovery; the need for renal replacement therapy and a composite endpoint of 30-day mortality, intensive care treatment or rehospitalization for respiratory distress.

### 3.2. Statistical Analyses

As this study was designed as a prospective cohort study enrolling consecutive patients presenting during the COVID-19 pandemic, no specific sample size was predetermined at the time of the study initiation All hypothesis testing was two-tailed, and a *p*-value < 0.05 was considered statistically significant. Discrete variables were expressed as counts (percentage) and continuous variables as medians and interquartile ranges (IQR). Comparisons between groups were made using the Kruskal–Wallis test and Pearson’s X^2^ test, as appropriate. A post-hoc analysis was performed using the Bonferroni method. We used Cox proportional hazard models for the time-to-event analysis. The proportional hazard assumption was tested using Schoenfeld residuals. We examined the association of AKI with the primary and secondary outcomes in two different models: In model 1, we adjusted for demographics (age and gender) and comorbidity burden using the predefined comorbidities (presence of coronary artery disease, congestive heart failure, arterial hypertension, obesity, diabetes mellitus, chronic pneumopathy, chronic kidney disease, chronic hepatopathy, rheumatic diseases, immunodeficiency, prior stroke and prior or active malignancy) expressed as a metric variable ranging from zero to twelve. In model 2, to account for disease severity, we adjusted the model using the National Early Warning Score (NEWS), which includes temperature, heart rate (HR), respiratory rate (RR), level of consciousness according to the alert, verbal, pain, unresponsive scale (AVPU), oxygen saturation and supplemental oxygen [10]. Missing values in the NEWS Score were imputed using chained equations in 10 datasets [11]. A regression analysis was performed on all 10 imputed datasets, and the results were pooled by applying Rubin’s rule. Patients with AKI but without COVID-19 were used as the reference group, if not otherwise stated. In the sensitivity analysis, we repeated our analysis with only hospitalized patients. The analyses were performed using R (R Core Team 2020. R: A language and environment for statistical computing. R Foundation for Statistical Computing, Vienna, Austria. URL https://www.R-project.org/, access date 22 June 2020).

## 4. Results

### 4.1. Patients and Demographics

**Figure 1:**
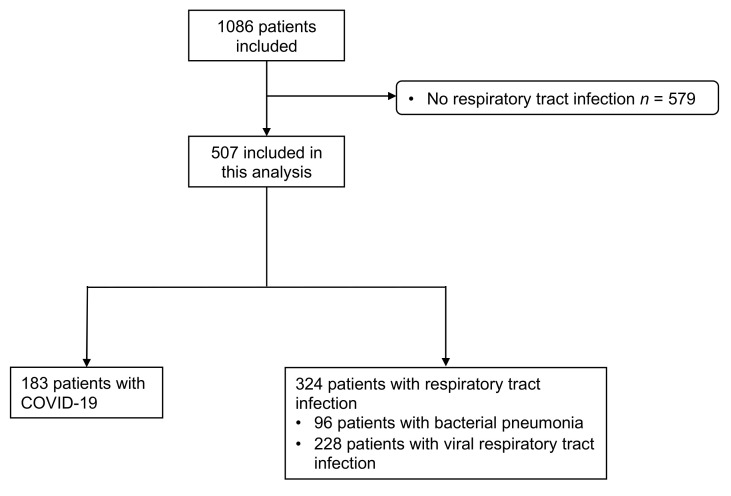
Flowchart of the study cohort. COVID-19 denotes coronavirus disease 2019.

Of the 1086 patients included in our study, 507 had an adjudicated diagnosis of a respiratory tract infection (Figure 1). Of those, 183 (36%) had a positive nasopharyngeal swap test for SARS-CoV-2. All other patients had the final adjudicated diagnosis of a viral (70%, *n* = 228) or bacterial infection (30%, *n* = 96) of the respiratory tract, including bronchitis, pneumonia and others. In patients with COVID-19, the median age was 57 years (interquartile range (IQR) 44, 69), and 44% were women. In patients without COVID-19, the median age was 58 years (IQR 42, 71, *p* = 0.775), and 44% were also women (*p* = 0.926). The proportion of patients with pre-existing pulmonary conditions (20% versus 39%, *p* < 0.001) was lower in patients with COVID-19. Laboratory sampling revealed lower concentrations of leukocytes (6.3 G/l (5.0, 8.3) versus 9.1 G/l (7.1, 11.7), *p* < 0.001), lymphocytes (1.0 G/l (0.7, 1.5) versus 1.6 G/l (1.0, 2.2), *p* = 0.001) and thrombocytes (216.0 G/l (174.2, 276.0) versus 249 G/l (206,293), *p* = 0.009) in patients with COVID-19. Other inflammatory markers, like c-reactive protein (CRP) (31.0 (3.5, 75.9) versus 9.0 (1.4, 48.0), *p* < 0.001) and ferritin (398 (167,841) versus 163 (85,299), *p* < 0.001), were increased to a higher level in patients with COVID-19 compared to patients without. The vital signs assessed at ED presentation were similar between patients with and without COVID-19. The median length of stay in patients with COVID-19 was longer compared to patients with other respiratory tract infections (7 days (IQR 4, 13) versus 6 days (IQR 3, 10), *p* = 0.006). The baseline demographic and clinical characteristics of all the patients are summarized in Table 1.

### 4.2. Incidence, Timing, Severity and Recovery of AKI

Overall, 95 patients (19%) developed AKI with a higher incidence in COVID-19 (55/183, 30%) compared to the controls (40/324, 12%, *p* < 0.001). The baseline characteristics of the patients with AKI stratified by COVID-19 status are presented in Table 2.

In the 55 patients with COVID-19 and AKI, 29 patients (53%) remained in AKI stage I compared to 26 patients (65%, *p* = 0.052) in those without COVID-19. Thirteen patients (24%) had stage II AKI among the patients with COVID-19 compared to nine patients (23%, *p* = 0.173) without COVID-19. Severe AKI was more frequent in COVID-19 patients (Thirteen patients (24%) versus five patients (13%), *p* = 0.009). There was no significant difference between the incidence of community-acquired AKI in patients with and without COVID-19 (54% versus 74%, *p* = 0.097). More patients with COVID-19 required intensive care unit admission compared to patients without COVID-19 (22% versus 7%, *p* < 0.001). The AKI incidence in patients in the intensive care unit was 78% in patients with COVID-19 compared to 61% in patients without COVID-19 (*p* = 0.264). Overall, eleven patients (2.2%; 13% of all AKI patients) required renal replacement therapy (RRT). The requirements for RRT were higher in patients with COVID-19 (eight patients (4.4%) versus three patients (0.9%); *p* = 0.03). Renal recovery during hospitalization was similar in patients with (35/55, 64%) and without COVID-19 (18/40, 45%, *p* = 0.110).

### 4.3. Predictive Value of AKI for Short-Term Mortality and Need for Intensive Care Treatment or Rehospitalization

In the overall cohort, after a median follow-up time of 30 days, 26 patients (5%) died, and 81 patients (16%) developed a composite endpoint of 30-day mortality, intensive care treatment or rehospitalization for respiratory distress. Thirty-day mortality was not significantly higher in those with COVID-19 compared to patients without COVID-19 (13/183, 7% versus 13/324, 4%, log-rank *p* = 0.100). Rehospitalization for respiratory distress was similar in both groups (COVID-19: 4/183, 2% versus 10/324, 3%, competing risk *p* = 0.709)

**Figure 2:**
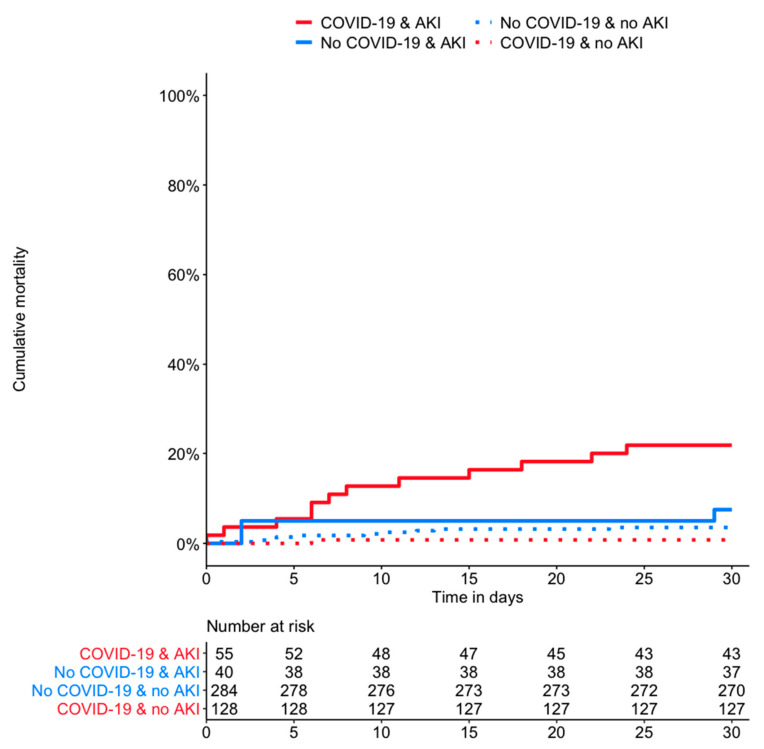
Cumulative 30-day mortality of patients with and without COVID-19 in the presence or absence of AKI. Solid lines represent patients with AKI, and dashed lines represent patients without AKI. Red lines represent patients with COVID-19 and blue lines patients with a respiratory tract infection without COVID-19. AKI denotes acute kidney injury.

In the overall population, the survival analysis showed an increased risk for 30-day mortality in patients with AKI compared to patients without (16% versus 3%, adjusted HR (aHR) 3.19, 95% confidence interval (CI) 1.39–7.33, *p* = 0.006, model 1). The risk of death was higher in patients with COVID-19-associated AKI compared to patients with AKI but without COVID-19 (aHR 3.98, 95% CI 1.10–14.46, *p* = 0.036, model 1, Figure 2). However, the association weakened when adjusting for disease severity (aHR 3.60, 95% CI 0.93–13.96, *p* = 0.062, model 2, Table 3).

Overall, the patients with AKI were at a higher risk of reaching the composite endpoint (aHR 8.87, 95% CI 5.28–14.90, *p* < 0.001). Additionally, the patients with COVID-19-associated AKI were at a higher risk compared to the patients with AKI but without COVID-19 (aHR 1.84, 95% CI 1.02–3.31, *p* = 0.042, model 1). This association persisted when adjusting for disease severity (aHR 2.35, 95% CI 1.29–4.25, *p* = 0.006, model 2, Table 3).

### 4.4. Sensitivity Analysis in Patients Requiring Hospital Admission

Overall, 63% of patients with COVID-19, compared to 42% without COVID-19, were admitted to the hospital. Of the patients admitted to the hospital, AKI occurred more frequently (45% versus 28%, *p* = 0.010) and with a numerically higher severity (26% stage 3 versus 13% stage 3, *p* = 0.136) in patients with COVID-19. Patients with COVID-19 and AKI were at the highest risk for death (aHR 3.78, 95% CI 1.05–13.63, *p* = 0.042, model 1). Again, this association weakened when adjusting for disease severity (aHR 3.71, 95% CI 0.96–14.31, *p* = 0.056, model 2).

## 5. Discussion

In this prospective analysis, we investigated the incidence, severity and associated outcomes of AKI in patients with COVID-19 compared to patients with other respiratory tract infections. We reported five major findings. First, AKI occurs more often in patients with COVID-19 compared to other respiratory tract infections. Second, AKI severity is higher in COVID-19 patients, with 4.4% of patients requiring acute RRT. Third, despite the higher incidence and more severe AKI, renal recovery is similar in patients with and without COVID-19. Fourth, compared to non-COVID-19 AKI, COVID-19-associated AKI is a risk factor for intensive care unit dependency, mortality and subsequent rehospitalization for respiratory failure. Fifth, the association of AKI with 30-day mortality weakens when adjusting for disease severity.

These results extend and corroborate previous studies establishing the important role and high burden of acute kidney injury in patients hospitalized with COVID-19 [3,4,5,6,12]. To the best of our knowledge, this is the first study to compare AKI prospectively in patients with COVID-19 in direct comparison to patients with other respiratory tract infections presenting consecutively with comparable symptoms during the same time period at the emergency department.

We found that the AKI incidence was significantly higher in patients with COVID-19, while the incidence of AKI in patients without COVID-19 was comparable to earlier studies investigating AKI in non-severe community-acquired pneumonia [13,14]. It has been previously shown that common AKI phenotypes like tubular necrosis or prerenal azotemia also occur in patients with COVID-19; however, additional AKI phenotypes unique to COVID-19 have been described recently [12,15]. For example, a renal tropism by SARS-CoV-2 using angiotensin-converting enzyme-2 as an entry receptor has been proposed and was confirmed in a large autopsy study isolating infectious SARS-CoV-2 from kidneys [16,17]. Additionally, a new form of a collapsing glomerulopathy has been described in individuals of African ancestry in the presence of a risk allele of the apolipoprotein L1 (APOL1) gene [18]. However, since most patients in our study were Caucasian, we do not believe that this contributed to the higher AKI rate in our study. The role of inflammatory cytokines in the development of acute kidney injury remains controversial. We found inflammatory markers such as CRP and ferritin to be significantly higher in patients with COVID-19; however, recent studies suggest that the cytokines measured in patients with COVID-19 are significantly lower compared to other diseases, such as severe acute respiratory syndrome (SARS) and Middle East respiratory syndrome (MERS) [19,20]. Although the occurrence of COVID-19-specific pathomechanisms might partially explain the higher incidence of AKI we observed, our analysis suggests that the higher incidence is mainly driven by the higher disease severity.

We found that AKI was more severe in patients with COVID-19, and additionally, more patients with COVID-19-associated AKI required renal replacement therapy. This is consistent with previous studies demonstrating a high incidence of severe and dialysis-dependent AKI in COVID-19 patients [3,4,21].

Importantly, we found the recovery rate of AKI to be similar in patients with and without COVID-19 when using a previously proposed definition of renal recovery and using patients with a respiratory tract infection as the control. The seemingly contrasting results by Fisher et al. [21] and Moledina et al. [22] were likely caused by the differing control groups and different definitions of renal recovery in these publications. This argument is strengthened further by additional studies describing similar recovery rates even in RRT-dependent AKI when using the same recovery definition [6,23,24]. The long-term consequences of COVID-19-associated AKI on renal and cardiovascular functions need to be explored in future studies.

In our analysis, AKI was associated with an excess in mortality among patients with COVID-19. This finding is in line with other studies demonstrating a higher mortality rate in patients with COVID-19-associated AKI [4,5,6]. However, the association in our study ablated when adjusting for disease severity. AKI is not only a known predictive factor for mortality but, also, a surrogate marker of severe diseases [25,26]. This strengthens the argument that the higher mortality observed in patients with COVID-19-associated AKI might also be partially explained by a higher general severity of the COVID-19 disease, while COVID-19 kidney-specific factors cannot be ruled out. A recently published analysis found the AKI rates to be higher in patients with COVID-19 compared to patients without after an adjustment for the known traditional risk factors of AKI, suggesting a direct effect [22]. However, we used patients with respiratory illnesses as the control that shared similar pathomechanisms of AKI contrary to using historical cohorts. Lastly, to some extent, the high mortality risk of COVID-19-associated AKI could also be attributed to the absence of a specific treatment for the underlying disease. Even more, potential nephrotoxic drugs were used to treat patients with COVID-19 in combination with a low amount of fluid resuscitation in the presence of or with impending acute respiratory distress syndrome, which might have also contributed to the high AKI incidence and the higher mortality in COVID-19.

## 6. Limitations

Some limitations merit consideration when interpreting the findings of this study. As urine sampling was not part of the study protocol, we did not have any information about proteinuria or albuminuria during the AKI episode to compare the different AKI phenotypes between both groups in more detail. Additionally, we did not have any information about the urine output for the diagnosis of acute kidney injury, which might have led to an underrepresentation of AKI. However, this has been the case in most previous studies on this topic, so our methodology is in line with the previously published works. As a result of a small sample size, adjustment of the statistical models for possible confounders was limited; however, by examining the different models and adjusting for the previously identified heavy confounders, we expected our results to be robust. Lastly, we were unable to account for potential nephrotoxic drugs or the specific COVID-19-related therapies that were used in the first wave of the COVID-19 pandemic.

In conclusion, AKI incidence and severity are higher and associated with worse outcomes in patients with COVID-19 as compared to other respiratory tract infections. This underlines the high burden of AKI during the COVID-19 pandemic and the need for the careful monitoring of renal functions in patients with COVID-19.

## Figures and Tables

**Table 1:** Baseline characteristics. CKD denotes chronic kidney disease, COPD denotes chronic obstructive pulmonary disease, ACE-Inhibitor denotes Angiotensin Converting Enzyme Inhibitor, ARB denotes angiotensin receptor blocker and News Score denotes National Early Warning Score. Values are the numbers (percentages) or median (interquantile range). To convert the creatinine values from μmol/L to mg/dL, divide by 88.4. Bold *p*-values represent significant differences.

Variable	Overall *n* = 507	COVID-19 *n* = 183	Non-Covid-19 *n* = 324	*p*-Value
Demographics				
Age, years	57 (43, 70)	57 (44, 69)	58 (42, 71)	0.775
Female	223 (44)	81 (44)	142 (44)	0.926
**Medical history**				
Valvular Cardiopathy	24 (5)	8 (4)	16 (5)	0.831
Coronary heart disease	64 (13)	21 (12)	43 (13)	0.656
Atrial fibrilation	42 (8)	9 (5)	33 (10)	**0.044**
Hypertension	223 (44)	81 (44)	142 (44)	0.926
Ever smoker	344 (68)	109 (60)	235 (73)	**0.003**
CKD	65 (13)	26 (14)	39 (12)	0.492
Intermittent hemodialysis	3 (1)	1 (1)	2 (1)	1.000
Diabetes	84 (17)	33 (18)	51 (16)	0.535
Obesity	164 (32)	73 (40)	91 (28)	**0.008**
Stroke	28 (6)	9 (5)	19 (6)	0.840
Hepatopathy	50 (10)	13 (7)	37 (11)	0.124
Cancer	47 (9)	17 (9)	30 (9)	1.000
Pneumopathy	164 (32)	37 (20)	127 (39)	**<0.001**
Asthma	79 (16)	25 (14)	54 (17)	0.444
COPD	67 (13)	9 (5)	58 (18)	**<0.001**
**Preadmission medication**				
ACE-Inhibitor	58 (11)	23 (13)	35 (11)	0.563
ARB	89 (18)	37 (20)	52 (16)	0.274
Diuretics	106 (21)	36 (20)	70 (22)	0.650
**Laboratory parameters at admission**				
Hemoglobin, g/L	139 (125, 150)	137 (128, 148)	140 (124, 150)	0.931
Leukocytes, 10^9^/L	8.2 (6.0, 10.7)	6.3 (5.0, 8.3)	9.1 (7.1, 11.7)	**<0.001**
Lymphocytes, 10^9^/L	1.4 (0.8, 2.0)	1.0 (0.7, 1.5)	1.6 (1.0, 2.2)	**<0.001**
Thrombocytes, 10^9^/L	236 (194, 288)	216 (174, 276)	249 (206, 293)	**<0.001**
C-reactive protein, mg/L	12.2 (1.8, 59.1)	31.0 (3.5, 75.9)	9.0 (1.4, 48.0)	**<0.001**
D-dimers, µg/mL	0.5 (0.3, 1.1)	0.6 (0.3, 1.2)	0.5 (0.3, 0.9)	**0.009**
Ferritin, µg/L	208 (97, 460)	398 (167, 841)	163 (85, 299)	**<0.001**
Creatine kinase, U/L	90.0 (56.8, 143.2)	85.5 (54.8, 152.5)	92.5 (58.0, 138.0)	0.921
eGFR, mL/min/1.73 m^2^	92 (70, 106)	92 (72, 108)	92 (66, 102)	0.062
Creatinine, µmol/L	74 (61, 91)	76 (62, 95)	72 (60, 89)	**0.044**
Urea, mmol/L	5.0 (3.7, 6.6)	5.0 (3.7, 6.2)	5.0 (3.8, 6.8)	0.754
Sodium, mmol/L	138 (135, 141)	137 (134, 140)	138 (136, 141)	**0.001**
Potassium, mmol/L	3.9 (3.7, 4.2)	3.9 (3.7, 4.2)	4.0 (3.7, 4.3)	0.128
LDH, U/L	221 (189, 284)	258 (204, 355)	209 (185, 252)	**<0.001**
**Physical exam at ED**				
Systolic BP, mmHg	137 (122, 153)	135 (122, 148)	139 (122, 155)	**0.036**
Diastolic BP, mmHg	82 (72, 89)	82 (71, 90)	82 (73, 89)	0.938
Heart rate, beats/min	90 (78, 103)	89 (80, 103)	90 (76, 104)	0.928
SpO2, %	97 (95, 98)	96 (94, 98)	97 (95, 98)	0.126
Respiratory Rate, breaths/min	20 (16, 24)	20 (16, 24)	19 (16, 23)	0.114
NEWS Score	3 (1, 5)	4 (2,6)	3 (1, 5)	**<0.001**

**Table 2:** Baseline characteristics of patients with acute kidney injury. AKI denotes acute kidney injury, CKD denotes chronic kidney disease, COPD denotes chronic obstructive pulmonary disease, ACE-Inhibitor denotes Angiotensin Converting Enzyme Inhibitor, ARB denotes angiotensin receptor blocker and News Score denotes National Early Warning Score. Values are the numbers (percentages) or median (interquantile range). To convert the creatinine values from μmol/L to mg/dL, divide by 88.4. Bold *p*-values represent significant differences.

Variable	Overall *n* = 95	COVID-19 + AKI *n* = 55	Non–Covid-19 + AKI *n* = 40	*p*-Value
**Demographics**				
Age, years	70 (60, 77)	68 (59, 77)	71 (63, 78)	0.388
Female	33 (35)	17 (31)	16 (40)	0.389
**Medical history**				
Valvular Cardiopathy	11 (12)	5 (9)	6 (15)	0.518
Coronary heart disease	29 (31)	14 (25)	15 (38)	0.302
Atrial fibrilation	15 (16)	6 (11)	9 (22)	0.158
Hypertension	69 (73)	39 (71)	30 (75)	0.816
Smoker	65 (68)	32 (58)	33 (82)	**0.014**
CKD	40 (42)	20 (36)	20 (50)	0.211
Intermittent hemodialysis	3 (3)	1 (2)	2 (5)	0.571
Diabetes	32 (34)	18 (33)	14 (35)	0.829
Obesity	47 (49)	32 (58)	15 (38)	0.062
Stroke	12 (13)	4 (7)	8 (20)	0.115
Hepatopathy	12 (13)	6 (11)	6 (15)	0.756
Cancer	13 (14)	7 (13)	6 (15)	0.770
Pneumopathy	30 (32)	15 (27)	15 (38)	0.372
Asthma	8 (8)	7 (13)	1 (2)	0.133
COPD	17 (18)	6 (11)	11 (28)	0.056
**Preadmission medication**				
Ace-Inhibitor	24 (25)	14 (25)	10 (25)	1.000
ARB	23 (24)	16 (29)	7 (18)	0.231
Diuretics	43 (45)	20 (36)	23 (57)	0.060
**Laboratory parameters at admission**				
Hemoglobin, g/L	133 (118, 148)	136 (126, 147)	125 (106, 148)	**0.043**
Leukocytes, 10^9^/L	9.2 (6.6, 13.8)	7.7 (5.8, 10.4)	11.1 (8.8, 16.8)	**<0.001**
Lymphocytes, 10^9^/L	0.8 (0.6, 1.2)	0.8 (0.6, 1.1)	1.0 (0.6, 1.3)	0.202
Thrombocytes, 10^9^/L	213 (146, 285)	200 (139, 247)	230 (166, 296)	0.270
C-reactive protein, mg/L	72.2 (32.1, 155.6)	76.2 (35.9, 154.5)	62.3 (16.8, 151.3)	0.399
D-dimers, µg/mL	1.2 (0.6, 3.5)	1.2 (0.7, 3.6)	1.3 (0.7, 3.2)	0.866
Ferritin, µg/L	500 (231, 1215)	861 (466, 1384)	274 (104, 435)	**<0.001**
Creatine kinase, U/L	134.0 (60.2, 330.0)	149.0 (70.5, 378.0)	105.0 (49.0, 229.0)	0.103
Creatinine, µmol/L	112 (84, 159)	106 (85, 151)	114 (85, 163)	0.930
Urea, mmol/L	8.7 (5.8, 13.9)	7.7 (5.7, 12.3)	9.1 (6.9, 14.6)	0.262
Sodium, mmol/L	136 (133, 139)	135 (133, 137)	136 (133, 141)	0.234
Potassium, mmol/L	4.1 (3.7, 4.5)	4.1 (3.6, 4.5)	4.2 (4.0, 4.5)	0.317
LDH, U/L	302 (225, 426)	358 (283, 461)	247 (208, 339)	**0.003**
**Physical exam at ED**				
Systolic BP, mmHg	129 (117, 148)	128 (119, 142)	130 (117, 156)	0.778
Diastolic BP, mmHg	71 (61, 86)	71 (63, 88)	76 (60, 85)	0.622
Heart rate, beats/min	90 (78, 108)	90 (78, 101)	100 (80, 112)	0.108
SpO2, %	94 (91, 96)	94 (93, 97)	94 (91, 96)	0.349
Respiratory Rate, breaths/min	23 (19, 28)	23 (16, 26)	25 (20, 30)	**0.031**
NEWS Score	7 (4, 10)	7 (5, 10)	6 (3,9)	**<0.001**

**Table 3:** Association of the AKI and COVID-19 status on mortality and the combined endpoint. Reference group was AKI in patients without COVID-19. Model 1 adjusted for age; sex and the presence or absence of twelve predefined comorbidities (presence of coronary artery disease, congestive heart failure, arterial hypertension, obesity, diabetes mellitus, chronic pneumopathy, chronic kidney disease, chronic hepatopathy, rheumatic diseases, immunodeficiency, prior stroke, prior or active malignancy). Model 2 adjusted for disease severity using the NEWS, which includes temperature, heart rate (HR), respiratory rate (RR), level of consciousness according to the AVPU, SPO2 and supportive oxygen. HR denotes hazard ratio; CI denotes confidence interval. Bolt p-values represent significant hazard ratios.

	HR	95% CI	*p*-Value
**30-day mortality**			
Unadjusted	3.09	0.87–10.96	0.080
Model 1	3.98	1.10–14.46	**0.036**
Model 2	3.60	0.93–13.96	0.062
**Combined endpoint**			
Unadjusted	1.74	0.98–3.12	0.059
Model 1	1.84	1.02–3.31	**0.042**
Model 2	2.35	1.29–4.25	**0.006**

## Data Availability

Restrictions apply to the availability of these data. The data presented in this study are available on reasonable request from the corresponding author.

## Supplementary Materials

The following are available online at https://www.mdpi.com/article/10.3390/jcm10112288/s1, Strobe Statement. Statistical supplement. Missing values.
