# Supplementary material for: Comparison of Acute Kidney Injury in Patients with COVID-19 and Other Respiratory Infections: A Prospective Cohort Study
Source: J Clin Med. 2021 May 25;10(11):2288. doi: 10.3390/jcm10112288 (PMC8197451; doi:10.3390/jcm10112288)
Supplement: Supplementary file 1 [file jcm-10-02288-s001.zip › jcm-1213291-supplementary.pdf]

Online-only supplement

Diebold M, Zimmermann T et al.

Table of contents

***Online-only supplement*....................1**
***STROBE Statement*....................2**
***Statistical Supplement*....................4**
***Missing values*....................5**
***Study Flowchart*....................7**

STROBE Statement

	Item No	Recommendation	Provided on page
**Title and abstract**	1	(*a*) Indicate the study's design with a commonly used term in the title or the abstract	1
		(*b*) Provide in the abstract an informative and balanced summary of what was done and what was found	3
**Introduction**			
Background/rationale	2	Explain the scientific background and rationale for the investigation being reported	4
Objectives	3	State specific objectives, including any prespecified hypotheses	4
**Methods**			
Study design	4	Present key elements of study design early in the paper	4
Setting	5	Describe the setting, locations, and relevant dates, including periods of recruitment, exposure, follow-up, and data collection	4-6
Participants	6	(*a*) Give the eligibility criteria, and the sources and methods of selection of participants. Describe methods of follow-up	4-5
		(*b*) For matched studies, give matching criteria and number of exposed and unexposed	NA
Variables	7	Clearly define all outcomes, exposures, predictors, potential confounders, and effect modifiers. Give diagnostic criteria, if applicable	7
Data sources/ measurement	8	For each variable of interest, give sources of data and details of methods of assessment (measurement). Describe comparability of assessment methods if there is more than one group	4-7
Bias	9	Describe any efforts to address potential sources of bias	7-8
Study size	10	Explain how the study size was arrived at	NA
Quantitative variables	11	Explain how quantitative variables were handled in the analyses. If applicable, describe which groupings were chosen and why	7-8
Statistical methods	12	(*a*) Describe all statistical methods, including those used to control for confounding	7-8
		(*b*) Describe any methods used to examine subgroups and interactions	7-8
		(*c*) Explain how missing data were addressed	8
		(*d*) If applicable, explain how loss to follow-up was addressed	7
		(*e*) Describe any sensitivity analyses	8
**Results**			
Participants	13	(a) Report numbers of individuals at each stage of study—eg numbers potentially eligible, examined for eligibility, confirmed eligible, included in the study, completing follow-up, and analysed	8, Figure S1

		(b) Give reasons for non-participation at each stage	Figure S1
		(c) Consider use of a flow diagram	Figure S1
Descriptive data	14	(a) Give characteristics of study participants (eg demographic, clinical, social) and information on exposures and potential confounders	8-9, Table 1 and 2
		(b) Indicate number of participants with missing data for each variable of interest	Table S1
		(c) Summarise follow-up time (eg, average and total amount)	10
Outcome data	15	Report numbers of outcome events or summary measures over time	8-11
Main results	16	(*a*) Give unadjusted estimates and, if applicable, confounder-adjusted estimates and their precision (eg, 95% confidence interval). Make clear which confounders were adjusted for and why they were included	10, Table 3
		(*b*) Report category boundaries when continuous variables were categorized	NA
		(*c*) If relevant, consider translating estimates of relative risk into absolute risk for a meaningful time period	NA
Other analyses	17	Report other analyses done—eg analyses of subgroups and interactions, and sensitivity analyses	10-11
**Discussion**			
Key results	18	Summarise key results with reference to study objectives	11
Limitations	19	Discuss limitations of the study, taking into account sources of potential bias or imprecision. Discuss both direction and magnitude of any potential bias	13-14
Interpretation	20	Give a cautious overall interpretation of results considering objectives, limitations, multiplicity of analyses, results from similar studies, and other relevant evidence	14
Generalisability	21	Discuss the generalisability (external validity) of the study results	12
**Other information**			
Funding	22	Give the source of funding and the role of the funders for the present study and, if applicable, for the original study on which the present article is based	15

Supplemental table S1 STROBE Statement Checklist

Statistical Supplement

List of used R packages:

- foreign
- haven
- tidyverse
- tableone
- flextable
- officer
- rms
- survminer
- openxlsx
- dplyr
- chisq.posthoc.test
- transplantr
- ggplot2
- scales
- mice
- cmprsk
-

Missing values

	Missing values n	Missing values %
Age	0	0
Female	0	0
Valvular Cardiopathy	0	0
Coronary heart disease	0	0
Atrial fibrilattion	0	0
Hypertension	0	0
Smoker	0	0
CKD	0	0
Dialysis	0	0
Diabetes	0	0
Obesity	0	0
Stroke	0	0
Hepatopathy	0	0
Cancer	0	0
Pneumopathy	0	0
Asthma	0	0
COPD	0	0
Ace-Inhibitor	0	0
ARB	0	0
Diuretics	0	0
Hemoglobin	14	3
Leukocytes	14	3
Lymphocytes	18	4
Thrombocytes	14	3
C-reactive protein	16	3
D-dimers	41	8
Ferritin	39	8
Creatinine kinase	15	3
Creatinine	14	3
Urea	16	3
Sodium	14	3
Potassium	14	3
LDH	46	9
NEWS Score	45	9
BD systolic	44	9
NEWS Score	45	9
BD diastolic	53	10
Hf	31	6
SpO2	27	5
Respiratory Rate	33	7

Supplemental Table S1 Missing values

Study Flowchart

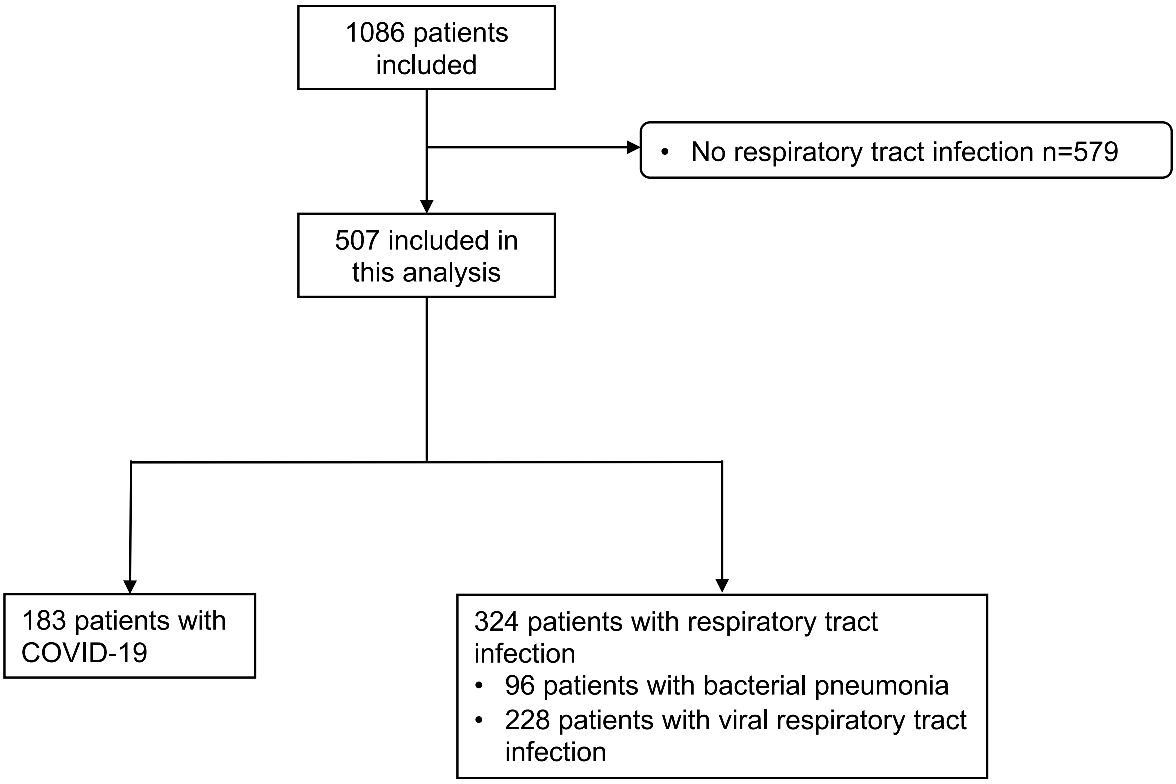

Figure S1 Study Flowchart
